# Disrupted Balance of Angiogenic and Antiangiogenic Signalings in Preeclampsia

**DOI:** 10.1155/2011/123717

**Published:** 2011-03-03

**Authors:** Mitsuko Furuya, Kentaro Kurasawa, Kiyotaka Nagahama, Kae Kawachi, Akinori Nozawa, Tsuneo Takahashi, Ichiro Aoki

**Affiliations:** ^1^Department of Pathology, Yokohama City University Graduate School of Medicine, Yokohama 236-0004, Japan; ^2^Department of Obstetrics, Yokohama City University Medical Center, Yokohama 232-0024, Japan; ^3^Department of Pathology, Yokohama City University Medical Center, Yokohama 232-0024, Japan

## Abstract

The placenta plays a central role in governing local circulatory system that mediates maternal condition and fetal growth. In early gestational phases, the placenta exerts properties of invasion and neovascularization for successful placentation. Extravillous invasive trophoblasts replace uterine endometrial vasculature and establish local blood pathway to obtain oxygen and nutrients from the mother. In later phases, the placenta promotes villous angiogenesis and vascular maturation that are finely controlled by angiogenic and antiangiogenic molecules. Among various molecules involved in placental neovascularization, vascular endothelial growth factor receptors (VEGFRs) and angiotensin II receptor type 1 (AT1) mediate important signaling pathways for maternal circulatory system and fetal growth. VEGFR1 and VEGFR2 are functional receptors for placental growth factor (PlGF) and VEGF, respectively, and PlGF-VEGFR1 and VEGF-VEGFR2 interactions are disturbed in many preeclamptic patients by excess amount of soluble form of VEGFR1 (also named sFlt1), a natural PlGF/VEGF antagonist. Recent studies have disclosed that excessive sFlt1 production in the placenta and aberrant AT1 signaling in the mother are closely associated with the pathology of preeclampsia and intrauterine growth restriction (IUGR). In this paper, neovascularization of the placenta and pathological events associated with disrupted balance between angiogenic and antiangiogenic signaling in preeclampsia are discussed.

## 1. Introduction

The placenta is a special organ that organizes fetal growth and maternal condition during gestation, and it terminates self-role as the fetomaternal mediator immediately after delivery. Pathological conditions during pregnancy such as preeclampsia and intrauterine growth restriction (IUGR) are closely associated with placental dysfunction. Maternal preeclamptic conditions frequently result in IUGR and premature delivery, and many studies on preeclampsia have improved our understanding of abnormal placentation in the context of shallow invasion and production of unfavorable proinflammatory factors. In the circulation of preeclamptic patients, some antiangiogenic molecules are detectable at excess levels [1–3], for example, soluble form of vascular endothelial growth factor (VEGF) receptor 1 (sVEGFR1, also named sFlt1) and soluble form of endoglin (sEng, also named sCD105). sFlt1 suppresses VEGF-mediated and placental growth factor- (PlGF-) mediated signaling, and sEng disturbs transforming growth factor *β*- (TGF*β*-) mediated signaling [2, 3]. These antiangiogenic cytokines are believed to be released from the placenta in response to hypoxic microenvironment. Once maternal vascular resistance increases, blood pressure *per se* potentially induces further dysfunctions such as glomerular endotheliosis and disruption of blood brain barrier. Therefore, spatiotemporal events that occur to the placenta and placenta-derived factors that induce maternal systemic dysfunction are very important for better understanding of pathological courses of preeclampsia and for better management of preeclamptic pregnancies. In this paper, we summarize current understanding of placental development and pathophysiology of preeclamptic placentas, with special attention on antiangiogenic signaling pathways.

## 2. Structure of Placental Vascular Network

**Figure 1:**
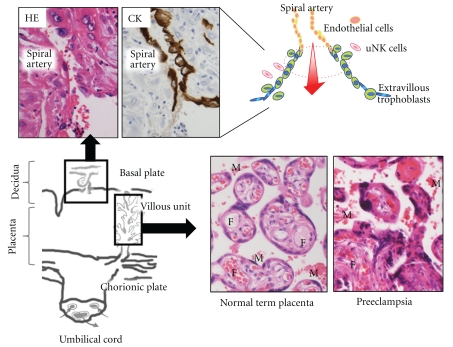
Schema and histology of the placenta. Endothelial cells of spiral arteries are replaced by cytokeratin- (CK-) positive extravillous trophoblasts (upper left). In terminal villous units, two distinct blood pathways exist, that is, maternal blood flow (M) and fetal circulation (F) that are separated by syncytiotrophoblasts. Villi are finely differentiated in normal term placentas, but poorly branched with fibrinous exudate and aggregation of syncytiotrophoblastic nuclei in preeclamptic placenta.

Human term placenta is divided largely into three layers in histology (Figure 1): (1) basal plate (maternal surface) and anchoring villi (most distal extensions of the primary stem villi) that interact directly with maternal endometrium, (2) terminal villous unit where gas and nutrient exchanges take place actively; (3) chorionic plate (fetal-side surface) and stem villi that consist of dense connective tissue containing larger fetal vessels. Amnion and chorion cover chorionic plate, and the umbilical cord collects chorionic arteries and veins on chorionic plate [4]. 

Fundamental structure of the placenta is established during the first half of gestation [5]. Terminal villous units (tertiary villi that stems from secondary villi) include fetal side capillaries lined by endothelial cells and outlining trophoblasts (Figure 1). In earlier stages, trophoblasts layer is composed of cytotrophoblasts (inner layer) and syncytiotrophoblasts (outer layer). As the pregnancy progresses, cytotrophoblasts layer become undetectable, and fetal capillaries are placed in close proximity to intervillous maternal circulation for maximizing gas exchange. Maternal blood space is lined directly by terminally differentiated syncytiotrophoblasts and not by endothelial cells, which is called hemochorial interface [6]. 

Fetal weights increase almost twice during the last stage. On the other hand, the weight of the placenta does not increase significantly in later stages [4]. Terminal villous vasculature becomes finely differentiated and vascular beds increase functional capacity of molecular exchanges between mother and fetus [4, 5] (Figure 1). In preeclamptic placenta, however, terminal villous units are poorly differentiated, and distal villi are truncated (Figure 1). These pathological changes are often accompanied by IUGR.

## 3. Pseudovasculogenesis and Local Microenvironment

Establishment of blood pathway is critical for successful placentation. At initial stages, extravillous trophoblasts infiltrate uterine implantation site and undergo invasive phenotype, remodeling endometrial tissue. Some of these trophoblasts exert vasculogenic property and replace endothelial cells of uterine spiral arteries (Figure 1). This process is called “pseudovasculogenesis” or “epithelial-endothelial transformation” [7]. Invasive endovascular trophoblasts temporarily form cellular plugs to restrict blood overflow during early period (6–12 weeks of gestation). The mechanism is not fully understood and some investigations argue against the importance of plugs. Certain local factors such as tumor necrosis factor *α* (TNF*α*) and TGF*β* may affect invasive properties of extravillous trophoblasts and alter susceptibilities of vascular constituent cells to trophoblasts-mediated stimuli [8]. Remodeled spiral arteries show characteristic feature of widened luminal diameter and degenerated vascular smooth muscle cells. As we discuss later, failure of vascular remodeling may lead to maternal preeclamptic conditions and IUGR. 

Transformed trophoblasts that replace uterine spiral arteries may express the endothelial markers such as CD31, VE-cadherin, vascular cell adhesion molecule (VCAM)-1 and *α*v*β*3 integrin [9, 10]. In addition, they potentially express endothelial nitric oxide synthase (eNOS), suggesting that they mimic endothelial cells morphologically and functionally [11]. Such phenotypic transformation is observed not only in placentation but also in tumor neovascularization. Some types of tumor cells may acquire endothelial cells-like features and form aberrant vascular network where endothelial lining is missing, known as “tumor vasculogenic mimicry” [12, 13]. These special tumor cells may also express some endothelial markers and vasculogenesis-related molecules such as VE-cadherin, CD34 and CD105 [13, 14]. Tumor invasion is disorganized whereas trophoblasts invasion is finely controlled by the crosstalk between endometrial components and extravillous trophoblasts. If the endometrium is ulcerated due to abortion or other inflammatory events, local immune system does not work properly and trophoblasts may aberrantly invade deep layer of uterine smooth muscle. Such conditions are called accrete, increate and percreta. 

Tissue-specific proinflammatory microenvironment is essential for proper placentation. Uterine natural killer (uNK) cells are major local resident immune mediators characterized by CD45^+^ CD69^+^ CD56^bright^ and CD16^−^ [15–17]. uNK cells are thought to play important roles in decidual reaction, remodeling of spiral arteries, and regulating invasive properties of trophoblasts [16, 18]. Invasive trophoblasts express unique repertoire of human leukocyte antigens (HLA)-C, HLA-E and HLA-G [16, 17, 19, 20]. Classical MHC class I molecules HLA-A and HLA-B that have polymorphism for initiating allograft rejection are not expressed in extravillous trophoblasts. [16]. uNK cells have both inhibitory and stimulatory surface receptors for regulating trophoblasts invasion [15, 19]. For example, HLA-E in trophoblasts interacts with NKG2 (CD94) in uNK cells [19], and attenuates cytotoxicity of uNK cells against invading trophoblasts. HLA-C in trophoblasts interacts with killer-cell immunoglobulin-like receptor (KIR)-family in uNK cells, and specific combination of maternal KIR and fetal HLA-C contributes to successful placentation [21], though the actual mechanism remains to be further investigated [22]. Hyperactivated uNK cells may produce high amounts of unfavorable cytotoxic factors such as granulysin and inhibit trophoblasts invasion by inducing apoptosis [23]. In addition to uNK cells, CD14^+^ CD68^+^ macrophages also participate in proinflammatory crosstalk between spiral arteries and extravillous trophoblasts. In basal plate, local macrophages produce TNF*α* that potentially induces trophoblasts apoptosis [24]. If these cytotoxic cytokines are overproduced by maternal-side immune cells, the case may result in miscarriage or shallow invasion.

## 4. Predisposing Factors of Preeclampsia

The onset of preeclampsia may depend not only on a sole or a few pathological events. It seems rather to be triggered by a load of various predisposing factors that induce circulatory disorders [25]. After the onset of hypertension, shear stress on vascular wall may lead to further deterioration of fetomaternal conditions. With regard to maternal genetic predisposition, special patterns of angiotensinogen gene variants and quantitative trait loci (QTL) on some chromosomes including AGT, STOX1, 5q, 10q and 13q QTL have been reported [26–29]. Maternal KIR-AA and fetal HLA-C2, but not fetal HLA-C1, lead to increased risk of preeclampsia [21]. Although the backgrounds and progression patterns of preeclampsia may vary among cases, it is widely accepted that poor placentation at early gestational stages is an important predisposing condition for disease development. Narrowed blood canals due to insufficient arterial remodeling make the placenta hypoxic, and in response, a series of proinflammatory factors are released from the placenta that damage maternal circulatory system. This process is composed of two stages, that is, poor placentation of early gestational period (stage I) and maternal systemic dysfunction in later period (stage II) [25].

## 5. Key Molecules Involved in the Pathophysiology of Preeclampsia

There are a wide variety of factors that potentially damage maternal blood vessels. Neurokinin-B, a family of peptides tachykinins, had been proposed to be a responsible molecule that would cause preeclampsia [30]. Although later studies did not fully approve the notion [31], a study demonstrated that neurokinin-B, with the help of thromboxane A2- (TXA2-) like molecule, suppressed angiogenic activities *in vitro* by downregulating VEGF, VEGFR1 and VEGFR2 in cultured endothelial cells [32]. Cumulative studies on preeclampsia have elucidated signaling crosstalks among PlGF/VEGF, RAS, and classic eicosanoids such as prostacyclin and TXA2. In this paper, we focus on some soluble factors that are thought to exert antiangiogenic properties in the circulation of preeclamptic patients.

### 5.1. Soluble Form of VEGFR1 (sVEGFR1, sFlt1)

sFlt1 is a natural soluble factor, and it is the truncated version of VEGFR1 that lacks transmembrane and intracellular signaling domains [33]. sFlt1 generally inhibits the signaling pathways of angiogenesis by binding to free forms of VEGF and PlGF [33, 34]. With regard to physiological and pathological conditions *in vivo*, sFlt1 is known to be essential for physiological avascularity in the cornea [35]. sFlt1 is also produced in some types of tumor tissues such as colorectal and breast cancers [36, 37]. In clinical studies on these tumors, the expression level of sFlt1 was correlated with favorable prognosis, probably owing to its antiangiogenic property. 

In the middle stage of gestation, free VEGF and PlGF levels in maternal circulation increase in normal pregnancy, but not in preeclamptic cases. On the other hand, circulatory sFlt1 levels in preeclamptic women are abnormally higher than those in normal controls [38–40]. Predominance of sFlt1 leads to systemic vascular dysfunction by interfering with homeostatic activities of VEGF and PlGF [33, 41–43]. Excess amount of sFlt1 is produced mainly by villous trophoblasts stimulated by the sera of preeclamptic patients, suggesting that certain maternal-side factor(s) such as agonistic autoimmune antibody against angiotensin II receptor type 1 (AT1) induce antiangiogenic signaling in syncytiotrophoblasts. Recently, involvement of another splice variant of sFlt1 has been reported [44, 45]. Sela et al. named it sFlt1-14 [45] and suggested that this alternative variant might be a predominant inhibitor of VEGF. Although contribution of sFlt1-14 for regulating PlGF-mediated signaling is a subject for future study, their studies indicate that more than one soluble factor affects VEGFRs properties in preeclamptic placentas.

### 5.2. PlGF and VEGFR1

Heterozygous VEGF^+/−^ embryos die due to vascular defects [46], whereas PlGF deficient mice are fertile with normal looking. Therefore, the roles of PlGF are not fully understood and this molecule is thought to be dispensable for embryonic vascular development in contrast to VEGF [47]. PlGF binds to VEGFR1 but not to VEGFR2 [48, 49], and VEGFR1^−/−^ mice died in utero with an overgrowth of endothelial cell-like abnormal cells [50]. Since the kinase activity of VEGFR2 is about ten-fold higher than that of VEGFR1 [51], actual roles of PlGF-VEGFR1 axis seem to be complex. Collecting the studies on various vascular diseases including tumor angiogenesis, it is likely that PlGF has both angiogenic and antiangiogenic properties depending on pathophysiological conditions. PlGF may displace VEGF from VEGFR1 and direct VEGF towards VEGFR2, accelerating angiogenesis [48]. On the other hand, PlGF/VEGF heterodimers potentially suppress angiogenesis induced by VEGF homodimers [52–54]. 

In pregnancy, plasma concentration of PlGF is elevated exponentially during middle stages (100–1000 pg/mL). The sFlt1 level is also matched to that of PlGF [40], whereas the VEGF level is around 5–10 pg/mL [55]. The considerable difference of mean expression levels between PlGF and VEGF indicates that PlGF may play a predominant role in fetoplacental development, and that circulating PlGF level may reflect pathological conditions of pregnancy as a sensitive marker [40, 55]. Tayade et al. suggested that local PlGF might accelerate functional maturation of uNK cells for the process of trophoblast invasion. In PlGF deficient mice, binucleate uNK cells abnormally increased in number, and smooth muscle layer of spiral arteries were thickened, indicating that the process of vascular remodeling was disturbed in some degree [56]. Although preeclamptic symptom was not reported in PlGF deficient mice, in human prospective study, PlGF is shown to be suppressed at first trimester before clinical symptoms become overt [57, 58]. The results suggest that PlGF upsurge is probably hampered by some predisposing factors at initial stage, and that low-PlGF milieu contributes to the development of preeclampsia as an early event rather than as a consequence of later stages.

### 5.3. Soluble Form of Endoglin (sCD105, sEng) and Other Soluble Factors

The concentrations of sEng (sCD105) are significantly elevated in preeclamptic patients, especially in severe cases named HELLP syndrome (Hemolysis, Elevated Liver enzyme, Low Platelets syndrome) [3]. CD105 is an auxiliary receptor for TGF*β*1 and TGF*β*3, and is expressed in various cell types including endothelial cells, vascular smooth muscle cells, and so on [3, 59, 60]. CD105 deficient mice embryos die at mid gestation due to poor vascular smooth muscle development [61]. In in vitro study using cultured endothelial cells, sEng (sCD105) was shown to inhibit capillary tube formation and attenuate vasodilation induced by TGF*β*1 and TGF*β*3 [3]. In the rat treated with sEng adenovirus, TGF*β*-mediated signaling was suppressed, leading to eNOS reduction and impaired vasodilation of renal microvessels. These results support the notion that CD105 is indispensable for embryonic neovascularization and that sEng inhibits TGF*β*-mediated vascular activities. On the other hand, in human trophoblasts, TGF*β*-CD105 axis seems to negatively regulate cellular activities [62]. The expression levels of HIF-1*α* and TGF*β*3 were upregulated under hypoxia in the early gestational stage, which attenuates invasive property of trophoblasts [63]. This signaling seems to explain in part shallow invasion that leads to later preeclamptic condition. Although experimental models and investigation methods are different, it should be carefully considered the roles of CD105 and sEng in physiological and pathological conditions of pregnancy in the context of gestational stages. Trophoblastic expression levels of TGF*β*3, CD105 and its soluble form may be considerably different depending on gestational periods [63, 64]. Fine balance of angiogenic and antiangiogenic signaling induction at appropriate time point seems to be very important for normal placental function. 

There are several important factors other than sFlt1 and sEng that participate in antiangiogenesis in preeclampsia, and some of them probably function in concert with VEGFRs-mediated and AT1-mediated signaling pathways. Serological studies in preeclampsia have elucidated the involvement of several soluble forms of adhesion molecules associated with leukocyte trafficking. These molecules include sVCAM-1 (soluble form of VCAM-1, also named sCD106), sE-selectin (sCD62E), sP-selectin (sCD62P) and sICAM-1 (also named sCD54). Most of them are reported to be elevated in preeclampsia, but the results are not always in agreement [65–67], which may be explained in part by different gestational periods for analysis. In breast cancer patients who were treated with VEGF inhibitor, plasma level of sVCAM-1 was reported to be elevated [68]. Since both preeclamptic patients and those who receive VEGF inhibitors are in the condition of disturbed physiological angiogenesis, these soluble factors are likely to reflect endothelial dysfunction. Maternal-side endothelial cells at basal plate express E-selectin and P-selectin, and invasive trophoblasts express cognate ligand of these selectins [69]. If increased sE-selectin and sP-selectin disturb cellular interaction at basal plate, they may lead to weakened placental anchorage to uterine wall. Further investigation is required to understand the mechanism of these soluble factors that potentially aggravate fetomaternal condition.

## 6. Role of Renin-Angiotensin System (RAS)

 RAS is a mastermind regulator for controlling blood pressure. In addition, RAS participates in a wide variety of biological activities including vascular remodeling, inflammation and tumor development [70–73]. AT1 is a principle G protein-coupled receptor (GPCR) for angiotensin II, and AT1 signaling leads to strong vascular contraction by activating several pathways including ERK and calcineurin [74, 75], and this activation induces hypertension, edema, proteinuria and so on [25]. 

Angiotensin II is not elevated in preeclamptic women, thus RAS was once thought to be unrelated to the pathogenesis of human preeclampsia. Later studies, however, disclosed aberrant activation of AT1-mediated signaling in preeclamptic patients [76, 77]. In normal pregnancy, maternal circulatory levels of renin and angiotensin II increase, but hypertension does not occur due to reduced sensitivity of AT1 to RAS [78]. On the other hand, in preeclampsia, angiotensin II is not increased but AT1-mediated signaling pathways are aberrantly activated. There seems to be at least two mechanisms that accelerate AT1 signaling, that is, the formation of AT1-bradykinin B2 heterodimers [76], and agonistic autoimmune antibody against AT1 (AT1-AA) [77]. As we have discussed, excess sFlt1 is believed to cause widespread maternal endothelial dysfunction by interfering with physiological PlGF and VEGF activities. Recent studies have demonstrated the close association between accelerated AT1 signaling and sFlt1 production [79, 80]. Stimulation of AT1 receptor of cultured trophoblasts using IgG from preeclamptic women resulted in the elevation of sFlt1* in vitro* [81]. Preeclamptic model mice with elevated RAS showed increased maternal plasma level of sFlt1* in vivo* [80]. 

Calcineurin is a calcium/calmodulin-dependent serine/threonine protein phosphatase, and it activates the transcription factor named nuclear factor of activated T cells (NFAT). NFAT-luciferase activity was significantly accelerated in Chinese hamster ovary (CHO) cells stimulated by IgG of a preeclamptic patient [81]. The overproduction of sFlt1 was successfully suppressed by calcineurin inhibitor FK506 or calcineurin siRNA in immortalized human trophoblasts cells [81]. These studies indicate that AT1-AA in maternal circulation potentially triggers calcineurin-NFAT transcriptional activities through AT1, and that the AT1-mediated GPCR signaling may disturb VEGFR-mediated receptor tyrosine kinase (RTK) signaling via calcineurin-NFAT in preeclampsia. The findings elucidate the signaling cascade from AT1 activation to VEGF suppression in preeclampsia. Spatiotemporal association of this cascade with poor placentation is a subject for future study. A recent study further demonstrated that AT1-AA was detectable in fetal cord blood of preeclamptic pregnancy, suggesting that maternal circulatory AT1-AA might also be available as a fetal-side marker for evaluating IUGR and other fetal condition [82].

## 7. Clinical Manifestations

Imbalance of angiogenic and antiangiogenic molecules and aberrant signaling cascades derange maternal circulatory system and then induce characteristic clinical symptoms including hypertension and proteinuria. We discuss typical clinical manifestations, and introduce some rodent models that cause preeclampsia-associated symptoms.

### 7.1. Hypertension and Proteinuria

Although preeclamptic patients show heterogeneous symptoms in the context of disease onset, severity, fetal growth rate and so on, hypertension and proteinuria are essential phenomena of this disease. Mechanism of increased vascular resistance and hypertension is explained in part by insufficient production of nitric oxide (NO) and prostacyclin (PGI2). NO works as a potent vasodilator, and angiogenic inhibitors such as sFlt1 and sEng suppress eNOS expression, which in turn reduces NO production and increases vascular resistance [83, 84]. PGI2, a member of classical eicosanoids, is another effective vasodilator. It is known that another eicosanoid TXA2 that induces vasoconstriction is overexpressed in preeclampsia [85] and that PGI2/TXA2 imbalance contributes to the development of preeclampsia [86, 87]. The damage of cardiomyocytes in human preeclampsia is estimated mainly by functional analysis, and histological information is limited. Studies of rodent models are informative for the analysis of these organs that are not available in human patients. In a study of RAS-induced preeclamptic mice, named pregnancy-associated hypertension (PAH) mice, the cardiac tissues at term showed severe damages of cardiomyocytes such as fibrosis and apoptosis in addition to hypertrophy [88]. Although aberrant RAS in this model may not be the case in human preeclampsia, the increase of sFlt1 in maternal blood due to accelerated AT1-mediated signaling [80] is common feature both in this model and human preeclampsia [89].

**Figure 2:**
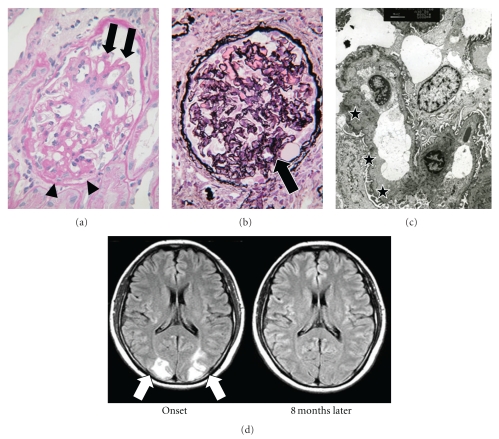
(a, b): A 40-year-old patient delivered a 736 g baby at 27 gestational weeks. Serum creatinine level was 2.06 mg/dl at 5 months after delivery. (a) A glomerulus shows segmental sclerosis and adhesion to Bowman's capsule (arrowheads). Glomerular capillary wall is thickened with double contour (arrows). (PAS, ×400). (b) A collapsing glomerulus revealing focal segmental sclerosis (arrow) with fibrinous exudate (PAS-methenamine silver, ×400). (c) A 41-year-old patient delivered a 2372 g baby at 37 weeks. Proteinuria prolonged for 9 months after delivery. Upon electron microscopy, subendothelial edema is observed (stars) (×3,000). (d) Diffusion abnormalities in a 32-year-old preeclamptic woman with RPLS. She complained of a headache from the beginning of labor at 37 weeks and lost her consciousness. Blood pressure was 181 mmHg and proteinuria was 8,900 mg/day. MRI illustrates the lesion of posterior lobes at the onset of convulsion (left, arrows). She recovered consciousness in a week, and the lesion diminished completely in 8 months after delivery (right).

Proteinuria may be induced not only by the increase of blood pressure but also by the disturbance of physiological vascular permeability. Under physiological condition, permeability of capillaries varies among organs and tissues. For example, cerebral capillaries are particularly impermeable, named blood brain barrier. On the other hand, capillary endothelial cells of renal glomeruli are characterized by fenestrate for fine traffic control of fluid and molecules. Depletion of VEGF from podocytes in a rodent model led to proteinuria and hypertension [90]. In this model, renal glomeruli were damaged by fibrin deposit and endotheliosis, suggesting that local effusion of physiological VEGF from podocytes toward endothelial cells is indispensable for maintaining fenestrated structure of glomerular vasculature [90]. Pregnant rats administrated adenovirus sFlt1 showed glomerular endotheliosis [3]. Such damages are also detectable in the kidneys of preeclamptic patients. The renal biopsies of severe preeclamptic patients showed diffuse glomerular endotheliosis, thickened capillary wall and focal sclerotic changes (Figures 2(a) and 2(b)). Electron microscopy highlighted subendothelial edema in glomerular capillaries (Figure 2(c)).

### 7.2. Reversible Posterior Leukoencephalopathy Syndrome (RPLS)

Other life-threatening complications of maternal-side include HELLP syndrome, pulmonary edema and eclampsia. Reversible posterior leukoencephalopathy syndrome (RPLS, also named posterior reversible encephalopathy syndrome) is the disorder of central nervous system associated with endothelial dysfunction in blood-brain barrier during and after pregnancies. Clinical and radiological features of RPLS were initially reported by Hinchey et al. in 1996 [91]. The most frequent cause of RPLS is believed to be hypertension, and the term is used not only for eclamptic condition but also for other endothelial dysfunction triggered by idiopathic hypertension, drug toxicity, systemic lupus erythematosus (SLE), and so on. Common manifestations of RPLS include headache, visual disturbance and seizure [92]. The lesion is clearly detectable by head MRI (Figure 2(d), left). RPLS is essentially curable without post-complications if treated properly based on anticonvulsion drugs and blood pressure control after delivery (Figure 2(d), right). Although many symptoms of eclampsia overlap with those of RPLS, a few cases of pregnancy-induced RPLS without preeclampsia have been reported [93, 94]. A reliable rodent model to analyze preeclampsia-associated RPLS has not been reported, yet, although PAH mice were reported to cause convulsion sometimes [95].

## 8. Summary

**Figure 3:**
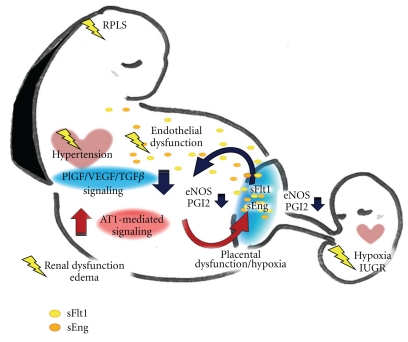
Schema of symptoms and signaling relationship between mother and placenta in preeclampsia. Aberrant AT1-mediated signaling in maternal vasculature and/or shallow invasion of trophoblasts makes the placenta hypoxic. In response, placental villous units produce sFlt1, sEng, and other proinflammatory cytokines that flow into maternal circulation, leading to systemic endothelial dysfunction. The increase of shear stress in fetoplacental site due to maternal hypertension further aggravates the whole system.

We have discussed the pathophysiology of preeclampsia from the points of antiangiogenic signaling pathways. Maternal susceptibility to unfavorable proinflammatory cytokines may vary among the cases; however, both pathogenetic factors produced by the placenta and responsive events in maternal circulation cooperatively develop fetomaternal disorders and may lead to life-threatening conditions (Figure 3). Apart from the studies on maternal systemic dysfunctions that have been intensively performed, very limited information is available about pathological events in fetoplacental-side, especially during later half of gestation as a fetus is indicated to grow exponentially. Current clinical information is obtained in most cases from maternal pathological data, and clinical evaluation of fetal status relies largely on ultrasonography and external tococardiography such as contraction stress test and nonstress test. Further information is necessary about the safety and effects of therapies on whole body in long-term prognosis. To this end, studies using animal models are necessary. Although rodent preeclamptic models may not mimic pathogenesis of human preeclampsia, time-course analysis of fetal body and the placenta will provide us with important information about pathological impacts on fetal well-being. A better understanding of the molecular and cellular crosstalks, proinflammatory microenvironment and the effects of antiangiogenic molecules will contribute to the improvement of effective and safe therapies for preeclampsia and for those suffering from vasculature diseases.
